# Anticoccidial Activity of Qinghao Powder Against *Eimeria tenella* in Broiler Chickens

**DOI:** 10.3389/fvets.2021.709046

**Published:** 2021-10-12

**Authors:** Ling Wang, Wenzhu Guo, Shahbaz Ul Haq, Zhiting Guo, Dongan Cui, Feng Yang, Feng Cheng, Xiaojuan Wei, Jiawen Lv

**Affiliations:** Key Laboratory of New Animal Drug Project, Gansu Province, Key Laboratory of Veterinary Pharmaceutical Development, Ministry of Agriculture and Rural Affairs, Lanzhou Institute of Husbandry and Pharmaceutical Sciences of Chinese Academy of Agriculture Sciences, Lanzhou, China

**Keywords:** *Eimeria tenella*, Qinghao Powder (QHP), recommended dose (RD), safety test, target animal

## Abstract

Artemisia annua (AAH) is traditionally used as an anti-malarial, expectorant and antipyretic Chinese medicine. The aim of this study was to explore the therapeutic effect of Qinghao Powder (QHP) on chicken coccidiosis, evaluate the safe dosage of QHP, and provide test basis for clinical medication. High-performance liquid chromatography (HPLC) and thin-layer chromatography (TLC) were used to detect artemisinin in Qinghao Powder (QHP) for quality control. The level of artemisinin in QHP was 81.03 mg/g. A total of 210 chicks (14 days of age) were divided randomly into seven groups: three QHP treatments (0.15, 0.30, and 0.60 g/kg), a toltrazuril control (1.00 mL/L), a sulfachloropyrazine sodium control (SSC, 0.30 g/L), an *E. tenella*-infected control, and a healthy control group. All the groups were inoculated orally with 7 × 10^4^ *E. tenella* oocysts except for the healthy control group. After seven days of administration, compared with the infected control group, chicks which were administered QHP, SS, and toltrazuril showed less bloody feces, oocyst output, and cecal lesions, and the protection rates were improved. The maximum rBWG and ACI were found in the SS-medicated group, followed by the groups medicated with 0.60 and 0.30 g/kg QHP. Therefore, a 0.30 g/kg dose level of QHP in the feed was selected as the recommend dose (RD) in the target animal safety test, in which 80 broiler chicks (14 days of age) were randomly divided into four major groups (I-healthy control group; II-1× RD; III-3× RD; IV-6× RD), with each group subdivided into two subgroups (A and B) consisting of 10 chicks each. After 7-day (for sub-group A) or 14-day (for sub-group B) administration, compared with the healthy control, treatment-related changes in BWG, feed conversion ratio (FCR), relative organ weight (ROW) of the liver, WBC counts, and levels of RBC, HGB, ALT, AST, and TBIL were detected in the 3× and 6× RD groups. No differences were noted in necropsy for all doses, and histopathological examinations exhibited no QHP-associated signs of toxicity or abnormalities in the liver or kidney. The findings suggest that QHP at a dose of 0.30 g/kg feed would be appropriate for therapy and intermittent treatment of *E. tenella*-infected chicks, the dosage in clinical applications should be set according to the recommended dose to ensure animal safety.

## Introduction

Chicken coccidiosis, a disease caused by apicomplexan protozoa of the genus *Eimeria*, is a significant problem in the poultry industry. There are seven *Eimeria* species affecting chickens, including *Eimeria acervulina, Eimeria brunetti, Eimeria maxima, Eimeria mitis, Eimeria necatrix, Eimeria praecox*, and *Eimeria tenella*. The parasites multiply in the intestinal epithelia, destroying the cells and reducing digestive capacity and nutrient absorption in the bird, with *Eimeria tenella* being highly pathogenic and causing caecal coccidiosis (1–5). Coccidiosis is responsible for 6–10% of all broiler mortalities, and the annual loss caused by *Eimeria* infection to the poultry industry is estimated at more than $3 billion (6, 7). At present, chicken coccidiosis has largely been controlled through the use of chemoprophylaxis and anticoccidial drugs added to feed, but there are complications due to the emergence of drug resistance and the toxic side effects of such additives on animal health (8–10). Increasing the development of drug-resistant coccidial species has stimulated the search for alternative control methods or new drugs, and this has become a top priority for the poultry industry (11–13). One alternative is the use of live virulent or attenuated vaccines or recombinant vaccines (14–16). However, live vaccines, particularly virulent ones, may have short-term adverse effects on chicken growth rate. Recombinant vaccines are still in the early stages of development. Therefore, until vaccines become more sophisticated, the use of anticoccidial drugs will continue.

*Artemisiae annuae herba* (AAH) is the dried aerial part of *Artemisia annua* L. [Asteraceae], a plant that has been traditionally utilized as an antimalarial, expectorant, and antifebrile compound in Chinese medicine. Moreover, AAH has anticoccidial properties when used alone or as the main herb in a complex formulation during treatment; however, as an animal coccidiostat, the content of the artemisinin within the crude extract of AAH or AAH complex has always been low, and the clinical use of artimisinin has been restricted due to the complexity of the synthetic route and the high synthetic cost. The findings from previous studies indicated that high doses of artemisinin can have adverse side effects such as neurotoxicity, renal toxicity, and cardiotoxicity in animals; the n-hexane extract of *Artemisiae annuae herba* at 0.50 g/kg of dose in feed reduced food intake and weight gain in chicks, and artemisinin administered continuously for 16 days at a high dose significantly inhibited the body weight gain of chickens (17–19). Qinghao Powder (QHP) prepared from the petroleum ether extract of the traditional Chinese medicine *Artemisiae annuae herba* has been shown to be effective against chicken coccidiosis (20, 21). As a plant-derived medicine, there are no scientific reports available concerning the content of active ingredients, anticoccidial activity, or safe dosage range. In the present study, high performance liquid chromatography (HPLC) and thin layer chromatography (TLC) were used to detect artemisinin in QHP for quality control; furthermore, a pathological model of chick coccidiosis was produced after *E. tenella* (Guangdong strain) was inoculated into chicks to determine the therapeutic effects and optimal recommended dose (RD) of QHP. The safe RD in target animals was further assessed for consequent clinical drug security according to the CVDE Guidelines (22). These experiments will provide a basis for the subsequent application of QHP and may assist in the approval of new animal drugs and preparations against chicken coccidiosis.

## Materials and Methods

### Drugs and Reagents

Petroleum ether, acetic ether, acetic acid, and n-hexane were purchased from Tianjin Chemical Reagent Company, China. Ethanol and xylene were purchased from Shanghai Chemical Reagent Company, China. Acetonitrile and methanol (HPLC grade) were purchased from Fisher Scientific (England); artemisinin was purchased from the National Institutes for Food and Drug Control (Purity of all ≥ 98%, China).

QHP (batch number: 20190520) was purchased from Heima Animal Pharmaceutical Co., Ltd., Henan Province, China. The positive control drug was toltrazuril (2.5%, w/v, batch number: 20190329, Bayer (Sichuan) Animal Health Co., Ltd., China). Sulfachloropyrazine sodium (SSC, 30%, w/w, batch number: 20190620) was purchased from Chongqing Yongjian Biotechnology Co., Ltd., China. Artemisinin from the petroleum ether extract of *Artemisiae annuae herba* was chosen to be a biomarker in TLC and HPLC evaluation for quality control.

### Thin Layer Chromatography Analysis (TLC)

As the active ingredient of *Artemisiae annuae herba*, the artemisinin in QHP was assayed by TLC according to the standardized experimental protocols of the Veterinary Pharmacopoeia of P. R. China (23). Petroleum ether (60–90°C)–acetic ether (4:5) was used as the developing solvent for artemisinin in silica gel–GF254 plates (Qingdao Haiyang Chemical Reagent Factory, China); 10% sulfuric acid ethanol solution containing 2% vanillin was used as the color developing reagent, and artemisinin was used as the standard preparation.

### High Performance Liquid Chromatography Analysis(HPLC)

The contents of artemisinin in QHP were determined by HPLC. Quantitative analysis of artemisinin in QHP was performed on the Agilent 1290 Infinity apparatus comprising two solvent delivery systems and a photodiode array detector (Agilent, USA). The column was an Agilent ZORBAX SB-C18 chromatographic column (4.6 mm × 250 mm, 5.0 μm). The mobile phase consisted of acetonitrile and H_2_O (60: 40), and the pH value was 6.8–7.2. Reagents were filtered through a Millipore 0.45 mm filter and degassed prior to use. The entire run was carried out by gradient elution at a flow rate of 1.0 mL/min; the detection wavelength was set at 210 nm; the column was maintained at 35°C, and the injection volume was 10 μL. Data collection and quantification were performed with Agilent Open LAB A.02.02 CDS ChemStation (Agilent, USA). The peak of artemisinin was identified by comparison with chemical standards.

### Ethics Statement

All of the experimental procedures were performed according to the principles of the Center for Veterinary Drug Evaluation (CVDE), Ministry of Agriculture, China (22). All of the animal experiments were conducted in strict accordance with the National Institutes of Health (NIH) Guidelines for the Care and Use of Laboratory Animals (24). All of the applicable international, national, and/or institutional guidelines for the care and use of animals were followed. The study was approved by the Ethics Committee of Lanzhou Institute of Husbandry and Pharmaceutical Sciences of the Chinese Academy of Agricultural Sciences (Approval No. LZMY 2020-016).

## Study Design

### *Eimeria tenella* Oocysts (Guangdong Strain)

The oocysts were isolated from chicks that had died from *E. tenella* infection in 1996 in Huadu, Guangdong Province, China, as confirmed by microscopic examination and sequence analysis of the rRNA gene internal transcribed spacer regions. The strain were maintained in the State Key Laboratory of Veterinary Etiological Biology, Lanzhou Veterinary Research Institute, CAAS. The oocysts were propagated in the broiler chicks without *E. tenella* infection by oral infection, and the feces were collected on post-infection (PI) days 6, 7, 8, and 9. The unsporulated oocysts were sporulated by placing in 2.5% K_2_Cr_2_O_7_ solution at suitable humidity and temperature (28°C). Sporulated oocysts were cleaned with water and counted by the McMaster technique described by Foreyt (25). The required concentration of the sporulated oocysts (70,000/mL) was maintained with phosphate buffered saline.

### Birds

A total of 290 one-day-old as-hatched Lingnan yellow-feathered broiler chicks (Lanzhou Hualong Commercial Hatchery) of both sexes were used. Chicks were reared under coccidia-free conditions and fed commercial food without coccidiostat additives for 14 days during the study (*ad libitum*). Chicks were reared under the following conditions: temperature (23 ± 2°C), relative humidity (55 ± 15%), and ventilation (air exchange rate of 18 cycles/h) (26).

### Anticoccidial Test

At 14 days of age (the day of challenge), the broiler chicks (*n* = 210) free from coccidian infection were weighed individually and randomly divided into seven treatments (1–7) with three pens containing 10 chicks each. Each pen was allocated to a large cage with a single tray per pen to catch the fecal material. All of the groups were inoculated orally with 7.0 × 10^4^ sporulated oocysts except for the healthy control group (1-HC). At 15 days of age, all of the chicks except for those in the healthy control (1-HC) and infected control group (2-IC) began drug treatment for 7 or 4 days (Table 1). At 22 days of age (post-inoculation day 8, PI day 8), after weighing the surviving chicks individually, all of the chicks were euthanized for the grading of cecal lesions, and the survival rate was calculated for each group. The clinical observations of bloody diarrhea and mortality for all the chicks were recorded daily throughout the experimental period. Treatments were as follows: 1-HC (healthy/negative control group; non-treated and non-infected); 2-IC (infected/positive control group; non-treated and *E. tenella*-infected); 3-TC (toltrazuril control, 1.00 mL/L water); 4-SSC (sulfachloropyrazine sodium control, 0.30 g/L water); 5-LG (low-dose group, 0.15 g/kg feed); 6-MG (middle-dose group, 0.30 g/kg feed); 7-HG (high-dose group, 0.60 g/kg feed).

**Table 1:** Effects of Qinghao Powder on bloody feces, oocyst output, and protection rate of chicks inoculated with *Eimeria tenella*.

**Groups/Drug concentration**	**No**.	**Treatments**	**Bloody feces^A^**				**Total blood feces**	**Oocyst output (× 10^6^)^B^**	**Protection rate (%)^C^**
		**Day 15 **~** Day 21**	**PI day 4**	**PI day 5**	**PI day 6**	**PI day 7**			
1-HCHealthy control	30	Feed without QHP and anticoccidial	0	0	0	0	0	0.00 ± 0.00^b^	100.00
2-ICInfected control	30	Feed without QHP and anticoccidial	3.6	4.0	4.0	4.0	15.6	17.62 ± 2.56^a^	0.00
3-TC1.00 mL/L toltrazuril	30	Feed with 1.00 mL/L of toltrazuril for 4 d	3.0	3.6	4.0	4.0	14.6	8.63 ± 1.95^bc^	51.02
4-SSC0.30 g/L of SS	30	Feed with 0.30 g/L of SS for 4 d	2.4	3.0	3.0	3.0	11.4	0.90 ± 0.32^b^	94.89
5-Low dose0.15 g/kg of QHP	30	Feed with 0.15 g/kg of QHP for 7 d	3.0	3.2	3.6	3.6	12.6	9.76 ± 2.38^bc^	44.60
6-Middle dose0.30 g/kg of QHP	30	Feed with 0.30 g/kg of QHP for 7 d	2.8	3.2	3.2	3.2	12.4	4.43 ± 1.52^bc^	74.85
7-High dose0.60 g/kg of QHP	30	Feed with 0.60 g/kg of QHP for 7 day	2.4	2.8	3.2	3.0	11.4	3.90 ± 1.37^bc^	77.86

*QHP, Qinghao powder; PI, post infection; HC, healthy control group (non-treated and non-infected); IC, infected control group (non-treated and E. tenella infected); TC, toltrazuril control; SS, sulfachloropyrazine sodium; SSC, sulfachloropyrazine sodium control*.

A *Bloody diarrhea score (median, IQR) of each group on PI day 4–7 after challenge with E. tenella*.

B *Means of three pens, data were presented as means ± SD*.

a, b, c *Columns with different superscripts present significant differences (P < 0.05)*.

C *Protection rate of each group on PI day 8. Protection rate (%) = (oocyst output of infected/unmedicated control group – oocyst output of medicated groups) ÷ (oocyst output of infected/unmedicated control group) × 100*.

Body weight gain (BWG) and survival rate (%): chicks from each treatment were weighed individually on day 14 (the day of inoculation) and day 22 (PI day 8). The individual and mean body weight gains were calculated for the period of days 14–22. The relative body weight gain (rBWG) and survival rate were calculated as follows:

BWG rate (%) = (final body weight – initial body weight) ÷ initial body weight × 100.

rBWG (%) = (BWG rate of the infected/unmedicated control or drug-treated group ÷ BWG rate of healthy control group) × 100.

Survival rate (%) = (number of surviving chicks in each group ÷ number of initial chicks in each group) × 100.

Fecal score and oocyst output in the feces: fecal droppings were examined visually for bloody diarrhea during 4–7 days PI and scored on a scale of 0–4 based on the evaluation standard of Suo and Li (27). Furthermore, on days 6, 7, and 8 after inoculation, the total daily fecal output of each pen of chicks was collected, and the daily oocyst production was determined using the McMaster technique (25, 28).

Cecal lesion score and oocyst value: on PI day 8, all the surviving chicks were euthanized, and the ceca were removed and opened. The infected ceca were examined and scored (from 0 to 4) according to the method described by Johnson and Reid (29). Lesion score = the average lesion score in each group ×10.

The cecal contents were ground, and the complete cecum contents of each chick were pooled. The total number of oocysts was determined from duplicate counts of diluted samples of homogenates using a hemocytometer counting technique (30). The results were expressed as OPG based on the method of JIAO (31).

Oocyst ratio = (OPG in healthy control or drug-treated group) ÷ (OPG in infected/unmedicated control group) × 100.

The anticoccidial index (ACI) established by Merk and Dohome (32) was calculated as ACI = (rBWG + survival rate) × 100 – (lesion score + oocyst value).

The total oocyst output per bird from feces and cecum was used to calculate the protection rate by the formula Protection rate (%) = (oocyst output of infected/unmedicated control group–oocyst output of healthy control or medicated groups) ÷ (oocyst output of infected/unmedicated control group) × 100 (14, 33, 34).

### Safety Test

This trial was conducted in line with the Guidelines on the Target Animal Safety Tests of Veterinary Traditional Chinese Medicines and Natural Medicines (22). Based on RD from the above-mentioned anticoccidial test, broiler chicks (*n* = 80, 14 days old) free from coccidian infection were selected as the target animals and were randomly divided into four major groups (I–IV) after weighing individually; each group was divided into two subgroups (A and B) consisting of 10 chicks each. At 14 days of age, chicks were fed with QHP at 1×, 3×, and 6× the recommended dose for 7 days (for the A sub-group) or 14 days (for the B sub-group). The groups were classified as I-A, I-B (healthy/negative control group, *ad libitum*); II-A, II-B (1× recommend dose, 1× RD); III-A, III-B (3× recommend dose, 3× RD); IV-A, IV-B (6× recommend dose, 6× RD). The behavior, feed intake, and deaths of the chicks were observed and recorded every day after administration. The weight on day 14 was considered as the initial weight. The feed conversion ratio (FCR) was calculated as grams of feed consumed to produce one gram of live weight. FCR was determined from the 3rd to 4th week of age.

On the 7th or 14th days after administration, the surviving chicks from subgroup A (only on the 7th day) or subgroup B (only on the 14th day) were individually weighted and euthanized. Blood was collected via cardiac puncture into EDTA-containing and non-heparinized tubes for hematological (automatic hematology analyzer, Sysme XT-1800i, China) and biochemical parameters assays (automatic blood biochemical detector, Olympus AU640, Japan). White blood cells (WBC), red blood cells (RBC), hemoglobin concentration (HGB), hematocrit (HCT, %), mean corpuscular hemoglobin concentration (MCHC), lymphocytes (Lym), monocytes (ML), alanine aminotransferase (ALT), aspartate aminotransferase (AST), total protein (TP), total bilirubin (TBIL), blood urea nitrogen (BUN), and creatinine (CRE) were measured.

After blood collection, all of the organs were examined and observed carefully, and macroscopic pathological changes were recorded. The selected organs (liver, kidney, spleen, heart, lung) were removed and weighed individually. The selected tissue samples (liver and kidney) were fixed with 10% buffered formalin solution and underwent routine histological processes for paraffin embedding and light microscopic examination. The relative organ weight (ROW) was calculated as organ weight (OW) as a percentage of body weight (BW).

### Statistical Analysis

The data were analyzed with the SPSS software program version 19.0 (IBM-SPSS Inc., Chicago, IL, USA). The bloody diarrhea score and lesion score of each group were compared by the non-parametric Kruskal-Wallis H test, and the results were presented as median (IQR, inter-quartile range). The parameters of oocyst output, body weight for each time point, body weight gains, relative organ weights (ROW), and biochemical and hematological indexes were analyzed by one-way ANOVA, followed by least significant difference (LSD) and Student's two-tailed *t*-test for the comparison between the test and control group, and Dunnett's test when the data involved three or more groups. Data are expressed as the mean ± standard deviations, *P*-values <0.05 (*P* < 0.05) were considered statistically significant.

## Results

### The Chemical Component Analysis of Qinghao Powder (QHP)

**Figure 1:**
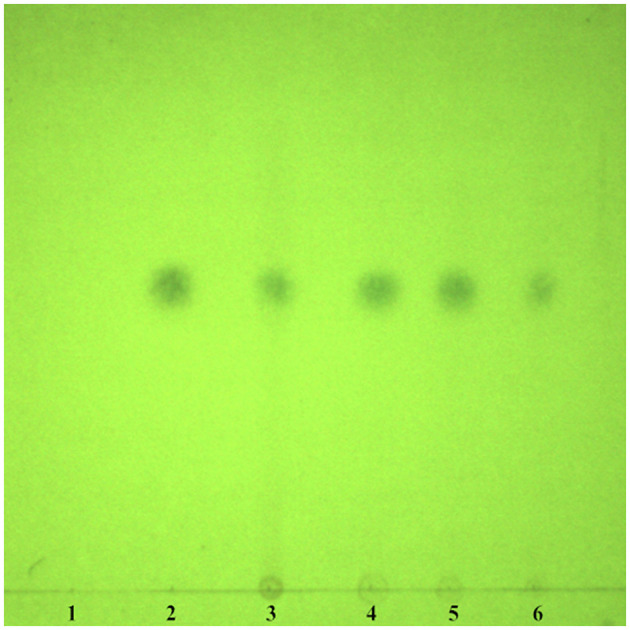
TLC chromatogram for detecting artemisinin in QHP. (1) Negative control. (2) Artemisinin. (3) Standard Chinese herbal medicine. (4-6) Samples of QHP.

**Figure 2:**
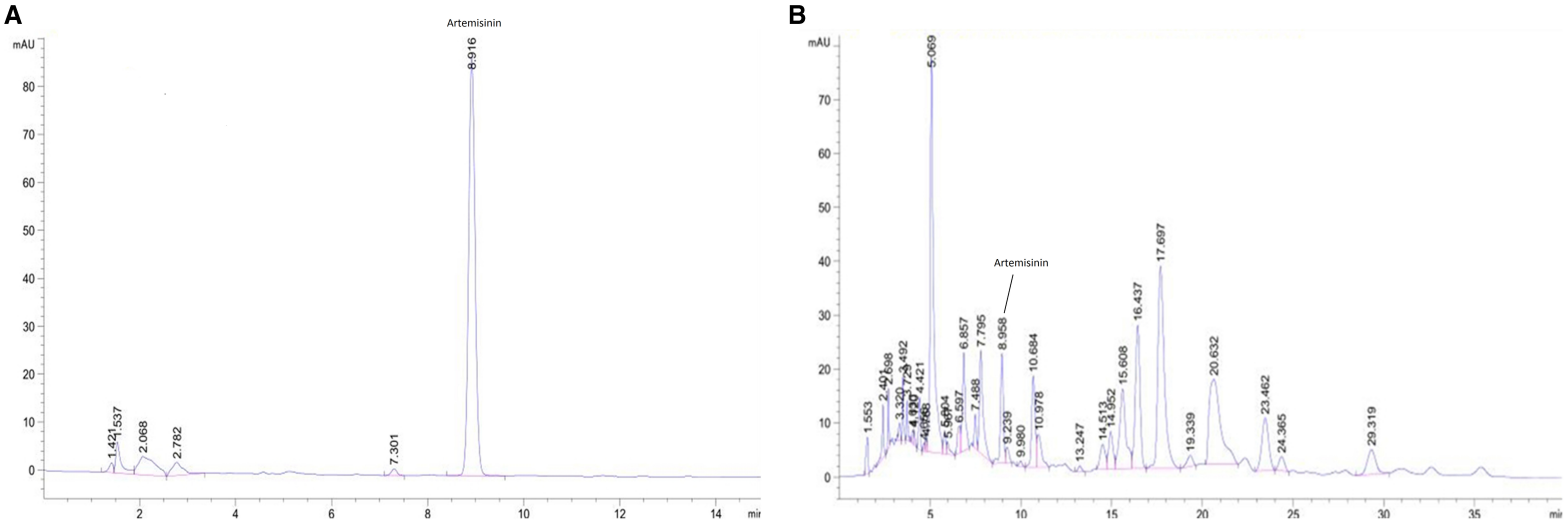
HPLC chromatogram for detecting artemisinin in QHP. **(A)** Artemisinin control. **(B)** Sample of QHP.

According to TLC analysis, artemisinin was present in QHP (Figure 1). As the active compound of *Artemisiae annuae herba* (AAH), artemisinin was present in QHP at 81.03 mg/g as per HPLC analysis (Figure 2).

### Anticoccidial Test

As shown in Tables 1, 2, bloody feces were observed in all infected groups after challenge, and 21 dead chicks were found during PI days 4–7. The oocyst output of groups administered with QHP, sulfachloropyrazine sodium, and toltrazuril were significantly less than that of infected control group (*P* < 0.05, *P* < 0.01). Furthermore, chicks treated with 0.30 g/L sulfachloropyrazine sodium and 0.60 g/kg QHP, which excreted less bloody feces and got higher protective rate among 5 infected groups. Among the drug medicated groups, the maximum rBWG was observed in the group administered with sulfachloropyrazine sodium (0.30 g/L water), followed by the groups administered with 0.30 g/kg feed QHP and 0.60 g/kg feed QHP. The lowest rBWG was observed in the group medicated with 1.00 mL/L toltrazuril.

**Table 2:** Effects of Qinghao Powder on the rBWG, lesion score, and ACI in chicks against *Eimeria tenella* (*n* = 30).

**Groups/Drug concentration**	**No**.	**Initial body weight (g)^A^**	**Final body weight (g)^B^**	**rBWG (%)^C^**	**Survival rate (%)^D^**	**Lesion score^E^**	**Oocyst value^F^**	**Anticoccidial index (ACI)^G^**
1-HCHealthy control	30	302.53 ± 29.61^a^	453.76 ± 20.85^a^	100	100	0	0	200
2-ICInfected control	30	303.68 ± 28.56^a^	404.65 ± 26.07^bc^	66.82	80.00	37.50	40	69.32
3-TC1.00 mL/L toltrazuril	30	297.53 ± 27.45^a^	382.33 ± 29.55^bc^	56.74	83.33	28.75	10	101.32
4-SSC0.30 g/L of SS	30	303.78 ± 27.88^a^	429.95 ± 34.01^a^	84.29	100.00	28.25	0	156.04
5-Low dose0.15 g/kg of QHP	30	302.92 ± 27.54^a^	406.13 ± 32.64^b^	68.87	80.00	29.50	20	99.37
6-Middle dose0.30 g/kg of QHP	30	306.59 ± 29.15^a^	420.58 ± 35.22^b^	75.75	86.66	28.25	5	129.16
7-High dose0.60 g/kg of QHP	30	298.55 ± 28.87^a^	417.96 ± 40.28^b^	75.30	100.00	26.25	5	144.05

*HC, healthy control group (non-treated and non-infected); IC, infected control group (non-treated and E. tenella infected); TC, toltrazuril control; SS, sulfachloropyrazine sodium; SSC, sulfachloropyrazine sodium control; BWG, body weight gain; rBWG, relative body weight gain*.

A, B *Data were presented as means ± SD*.

a, b, c *Values with different superscripts in the same column differ significantly (P < 0.05)*.

C *rBWG (%) = (BWG of the infected/unmedicated control or drug-treated group ÷ BWG of healthy control) × 100*.

D *Survival rate (%) = (number of surviving chicks in each group ÷ number of initial chicks in each group) × 100*.

E *Lesion scores (median, IQR) of cecum examined on the PI day 8*.

F *Oocyst value of each group on PI day 8. Oocyst value = 0 (an oocyst ratio of 0–1%); oocyst value = 5 (an oocyst ratio of 1–25%); oocyst value = 10 (an oocyst ratio of 26–50%); oocyst value = 20 (an oocyst ratio of 51–75%); and oocyst value = 40 (an oocyst ratio of 76–100%); Oocyst ratio = (OPG in healthy control or drug-treated group) ÷ (OPG in infected/unmedicated control group) × 100%; OPG, oocyst per gram*.

G *Anticoccidial index (ACI) of each group. ACI = (rBWG + survival rate) × 100 – (lesion score + oocyst value)*.

Chicks in the infected/non-treated group (2-IC) displayed the most severe swelling in the cecum. Cecal lesions were also found in all chicks in other infected groups, and the degree of damage was lower than that of the infected control group. No obvious lesions in other organs were found in all groups. Chicks in the five drug-treated groups showed a reduction in oocyst production; the maximum reduction was found in the sulfachloropyrazine sodium medicated group followed by the groups medicated with 0.60 g/kg QHP and 0.30 g/kg QHP. The anticoccidial activity in the sulfachloropyrazine sodium and QHP (at doses of 0.60 g/kg feed and 0.30 g/kg feed) treatments were superior to toltrazuril treatment in terms of oocyst output, protection rate, rBWG, cecal damage, and ACI values.

### Safety Test

**Figure 3:**
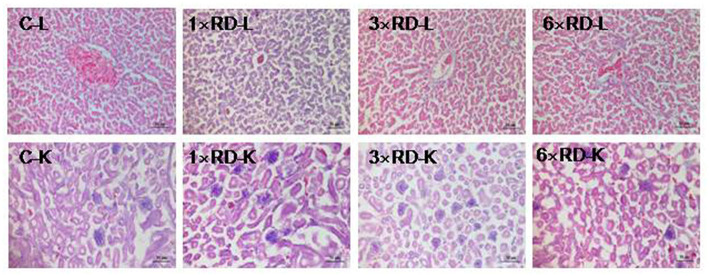
Histopathological analysis of organs in the control (C) and three QHP-treated groups (1× recommend dose, 1× RD; 3× recommend dose, 3× RD; 6× recommend dose, 6× RD) after 7-day administration (H&E stained); livers (L, 100×); kidney (K, 100×). Scale bar = 50 μm.

**Figure 4:**
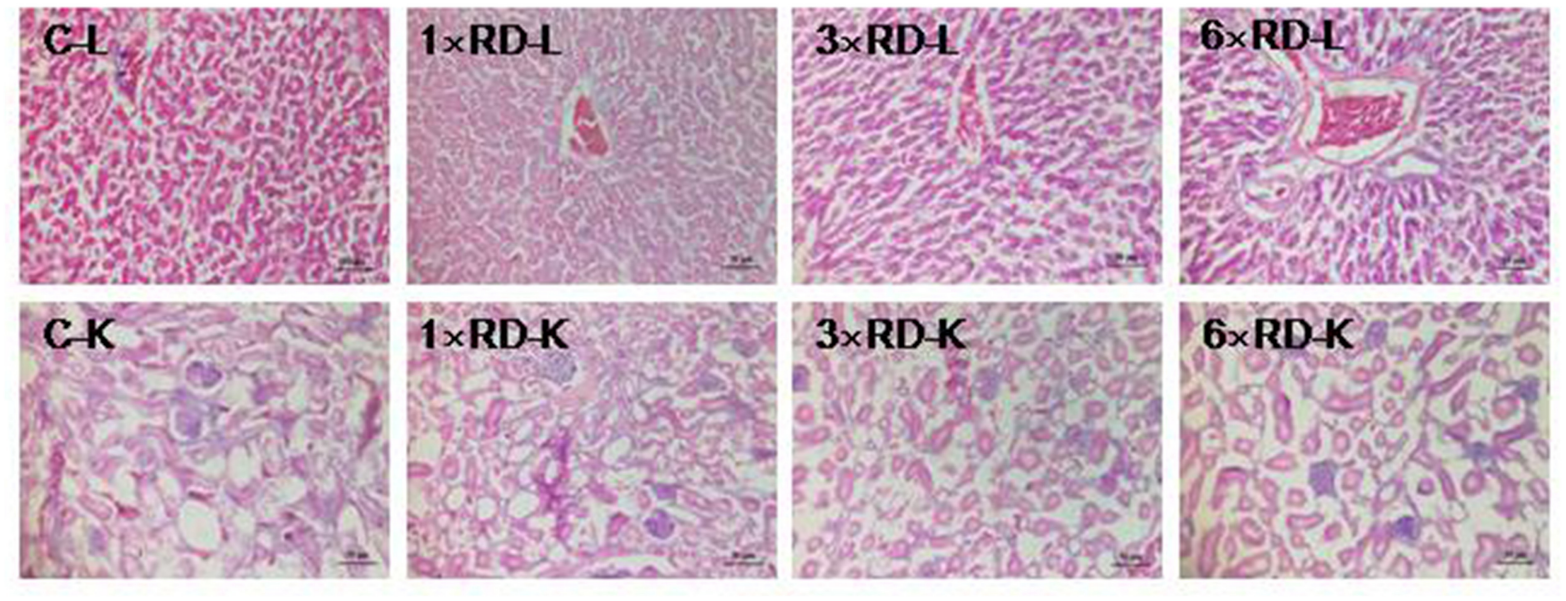
Histopathological analysis of organs in the control (C) and three QHP-treated groups (1× recommend dose, 1× RD; 3× recommend dose, 3× RD; 6× recommend dose, 6× RD) after 14-day administration (H&E stained); livers (L, 100×); kidney (K, 100×). Scale bar = 50 μm.

No deaths or abnormal changes in behavior, clinical condition, or feed intake occurred in chicks during the experimental period. There were no visible pathological changes in the heart, liver, lung, spleen, kidney, or other organs 7 or 14 days after administration. Histopathological examination of the liver and kidney revealed no abnormal pathological lesions in the QHP-treated chicks compared with the controls, as shown in Figures 3, 4.

Compared with the control, after 7- or 14-day administration, there were significant differences in the body weight gain (BWG) of chicks in 3× or 6× RD groups (*P* < 0.05, *P* < 0.01); moreover, lower rBWG and higher feed conversion ratios (FCR) were exhibited in these two groups at 14th day after administration, but similar BWG and lower FCR were observed in the 1× RD group (Table 3). The results of ROWs showed that there were no significant differences between the chicks in three dose groups of QHP and the control group after 7 or 14 days of administration (except for ROW of liver in the 6× RD group at 14th day after administration, *P* < 0.05) (Table 4). Furthermore, no significant differences were observed in any of the hematological/biochemical indexes of the two QHP-treated groups (3× RD and 1× RD) after 7 days of administration; while the total counts of RBC and WBC, as well as the levels of ALT, AST, and TBIL in the 6× RD group, which was significantly different to the control at 7th or 14th day after administration (*P* < 0.05) (Table 5).

**Table 3:** Effect of Qinghao Powder on growth performance of chicks in the safety test (*n* = 10).

**Treatments**	**Initial BW (g)^A^**	**Final BW (g)^B^**	**BWG (g)^C^**	**rBWG (%)^D^**	**Feed intake (g)^E^**	**FCR^F^**
**7th day after administration**						
Control	238.87 ± 12.83^a^	387.59 ± 13.03^a^	142.75 ± 20.37^a^	100	245.30	1.718
1× RD (0.30 g/kg)	241.26 ± 12.28^a^	389.57 ± 12.54^a^	143.34 ± 19.79^a^	100.41	257.69	1.798
3× RD (0.90 g/kg)	242.45 ± 14.79^a^	402.54 ± 15.06^a^	157.17 ± 13.28^b^	110.10	279.56	1.779
6× RD (1.80 g/kg)	241.60 ± 12.53^a^	399.08 ± 16.99^a^	151.42 ± 18.95^b^	106.07	288.07	1.902
**14th day after administration**						
Control	238.87 ± 12.83^a^	547.35 ± 22.84^a^	301.58 ± 21.36^a^	100	545.26	1.808
1× RD (0.30 g/kg)	241.26 ± 12.28^a^	546.77 ± 21.67^a^	300.60 ± 20.36^a^	99.68	537.60	1.788
3× RD (0.90 g/kg)	242.45 ± 14.79^a^	485.30 ± 20.70^bc^	237.95 ± 24.38^bc^	78.90	537.45	2.259
6× RD (1.80 g/kg)	241.60 ± 12.53^a^	464.79 ± 21.08^bc^	216.11 ± 22.25^bc^	71.66	529.17	2.449

*RD, recommend dose; BWG, body weight gain; rBWG, relative body weight gain; FCR, feed conversion ratio*.

A−C *Data were presented as means ± SD*,

a, b, c *Values with different superscripts in the same column differ significantly (P < 0.05)*.

D *rBWG (%) = (BWG of the drug-treated group ÷ BWG of control group) × 100*.

E *The feed intake of each group was measured by subtracting the residual feed weight from the offered feed weight during the trial*.

F *FCR was calculated as grams of feed consumed to produce one gram of live weight. FCR = feed intake (g)/BWG (g)*.

**Table 4:** Effect of Qinghao Powder on the relative organ weight (ROW) of chicks in the safety test (*n* = 10).

**Indexes**	**Groups and treatments (g/kg)**			
	**Control**	**1× RD (0.30 g/kg)**	**3× RD (0.60 g/kg)**	**6× RD (1.80 g/kg)**
**7th day after administration**				
Heart^A^	0.75 ± 0.08^a^	0.72 ± 0.05^a^	0.74 ± 0.09^a^	0.75 ± 0.10^a^
Liver^B^	2.52 ± 0.06^a^	2.48 ± 0.07^a^	2.44 ± 0.08^a^	2.46 ± 0.09^a^
Spleen^C^	0.13 ± 0.05^a^	0.12 ± 0.06^a^	0.14 ± 0.08^a^	0.11 ± 0.03^a^
Lung^D^	0.67 ± 0.09^a^	0.67 ± 0.10^a^	0.69 ± 0.04^a^	0.71 ± 0.05^a^
Kidney^E^	0.83 ± 0.07^a^	0.82 ± 0.06^a^	0.82 ± 0.07^a^	0.84 ± 0.10^a^
**14th day after administration**				
Heart^A^	0.64 ± 0.13^a^	0.63 ± 0.10^a^	0.65 ± 0.11^a^	0.66 ± 0.09^a^
Liver^B^	2.65 ± 0.12^a^	2.63 ± 0.08^a^	2.63 ± 0.13^a^	2.52 ± 0.10^b^
Spleen^C^	0.11 ± 0.05^a^	0.13 ± 0.04^a^	0.13 ± 0.06^a^	0.12 ± 0.07^a^
Lung^D^	0.71 ± 0.16^a^	0.68 ± 0.21^a^	0.71 ± 0.18^a^	0.69 ± 0.19^a^
Kidney^E^	0.82 ± 0.20^a^	0.83 ± 0.17^a^	0.82 ± 0.15^a^	0.81 ± 0.21^a^

A−E *Data were presented as means ± SD*.

a, b *Values with different superscripts in the same row differ significantly (P < 0.05)*.

**Table 5:** Effects of Qinghao Powder on hematological and serum biochemical indexes of chicks in the safety test (*n* = 10).

**Indexes**	**Groups and treatments (g/kg)**			
	**Control**	**1× RD (0.30 g/kg)**	**3× RD (0.60 g/kg)**	**6× RD (1.80 g/kg)**
**7th day after administration**				
RBC (× 10^12^/L)^A^	2.53 ± 0.10^a^	2.47 ± 0.26^a^	2.53 ± 0.25^a^	2.59 ± 0.30^a^
HGB (g/L)^B^	94.60 ± 5.82^a^	95.60 ± 7.88^a^	97.00 ± 6.86^a^	98.60 ± 7.74^a^
HCT^C^	32.06 ± 2.15^a^	31.04 ± 2.25^a^	31.12 ± 2.91^a^	32.45 ± 2.37^a^
MCHC (g/L)^D^	270.00 ± 20.51^a^	272.00 ± 27.38^a^	275.80 ± 28.01^a^	280.00 ± 27.43^a^
WBC (×10^9^/L)^E^	253.10 ± 18.24^a^	256.26 ± 15.32^a^	259.14 ± 23.50^a^	254.50 ± 20.33^a^
Lym (×10^9^/L)^F^	10.50 ± 1.22^a^	10.96 ± 1.18^a^	11.22 ± 1.23^a^	10.80 ± 1.25^a^
ML^G^	1.13 ± 0.20^a^	1.16 ± 0.22^a^	1.28 ± 0.25^a^	1.23 ± 0.28^a^
ALT (U/L)^H^	2.05 ± 0.23^a^	2.12 ± 0.25^a^	2.30 ± 0.36^a^	2.60 ± 0.88^b^
AST (U/L)^I^	230.40 ± 15.24^a^	237.07 ± 19.60^a^	238.40 ± 17.15^a^	250.60 ± 14.80^b^
TP (g/L)^J^	32.70 ± 2.12^a^	33.54 ± 2.36^a^	36.80 ± 7.24^a^	37.20 ± 2.58^a^
TBIL (μmol/L)^K^	9.68 ± 1.20^a^	9.62 ± 1.10^a^	9.70 ± 1.36^a^	10.80 ± 1.06^b^
BUN (mmol/L)^L^	0.77 ± 0.12^a^	0.70 ± 0.15^a^	0.70 ± 0.18^a^	0.61 ± 0.16^a^
CRE (μmol/L)^M^	10.54 ± 0.70^a^	10.58 ± 0.97^a^	10.66 ± 0.90^a^	10.70 ± 1.06^a^
**14th day after administration**				
RBC (×10^12^/L)^A^	2.44 ± 0.20^a^	2.47 ± 0.18^a^	2.51 ± 0.22^a^	2.25 ± 0.17^b^
HGB (g/L)^B^	98.43 ± 7.45^a^	99.4 ± 9.60^a^	97.80 ± 8.96^a^	88.40 ± 10.25^b^
HCT^C^	31.62 ± 2.20^a^	30.23 ± 2.30^a^	32.05 ± 2.18^a^	31.28 ± 2.16^a^
MCHC (g/L)^D^	278.40 ± 25.38^a^	272.60 ± 23.59^a^	268.95 ± 25.67^a^	270.40 ± 26.55^a^
WBC (×10^9^/L)^E^	264.22 ± 24.35^a^	260.34 ± 26.50^a^	259.52 ± 24.38^a^	276.04 ± 27.10^b^
Lym (×10^9^/L)^F^	10.20 ± 1.26^a^	10.48 ± 1.25^a^	10.36 ± 1.17^a^	10.51 ± 0.93^a^
ML^G^	1.14 ± 0.41^a^	1.25 ± 0.32^a^	1.14 ± 0.27^a^	1.26 ± 0.86^a^
ALT (U/L)^H^	2.10 ± 0.22^a^	2.11 ± 0.24^a^	2.18 ± 0.23^a^	2.20 ± 0.28^a^
AST (U/L)^I^	232.80 ± 12.45^a^	233.40 ± 12.69^a^	235.60 ± 14.23^a^	240.15 ± 17.62^a^
TP (g/L)^J^	30.14 ± 2.59^a^	33.21 ± 2.48^a^	32.48 ± 2.82^a^	32.67 ± 3.50^a^
TBIL (μmol/L)^K^	9.58 ± 1.20^a^	9.60 ± 1.08^a^	9.95 ± 1.36^b^	10.50 ± 1.98^b^
BUN (mmol/L)^L^	0.75 ± 0.12^a^	0.78 ± 0.15^a^	0.79 ± 0.10^a^	0.80 ± 0.15^a^
CRE (μmol/L)^M^	10.64 ± 1.10^a^	10.69 ± 1.02^a^	10.72 ± 1.03^a^	10.85 ± 1.26^a^

*RBC, red blood cells; HGB, hemoglobin concentration; HCT, hematocrit; MCHC, mean corpuscular hemoglobin concentration; WBC, white blood cell; Lym, lymphocyte; ML, monocytes; ALT, alanine aminotransferase; AST, aspartate aminotransferase; TP, total protein; TBIL, total bilirubin; BUN, blood urea nitrogen; CER, creatinine*.

A−M *Data were presented as means ± SD*.

a, b *Values with different superscripts in the same row differ significantly (P < 0.05)*.

## Discussion

*Artemisiae annuae herba* (AAH) exhibits good clinical efficacy in treatments as an antimalarial, expectorant, or antifebrile agent in Chinese traditional medicine. In addition, artemisinin exerts control effects against toxoplasmosis, chicken coccidia, schistosomiasis, eperythrozoonosis, and *Pneumocystis carinii* infection (17, 18, 35). However, preclinical studies such as animal acute/chronic toxicity experiments revealed that high doses or long-term exposure to artemisinin can have toxic effects in multiple systems and organs (17–19). Therefore, QHP prepared from the petroleum ether extract of *Artemisiae annuae herba* required a deeper evaluation of its efficacy and safety concerning its anticoccidial properties prior to clinical applications. Control of the quality of medicinal materials and preparations with modern analytical tools is important to ensure their efficacy. In this study, artemisinin in QHP was identified and assayed using HPLC and TLC. The results showed that artemisinin was present in QHP, and the content of artemisinin in QHP was 81.03 mg/g. We can make a preliminary conclusion that the quality of QHP in terms of artemisinin content remains acceptable.

In this study, supplementation of QHP, sulfaclopyrazine sodium (SSC), and toltrazuril in feed alleviated the signs of infection. After seven days of administration, the number of oocysts significantly decreased (*P* < 0.05) after treatment of the infected chicks with QHP, SS, and toltrazuril. The protection rate for groups 4-SSC (0.30 g/L of SS), 6-middle dose (0.30 g/kg of QHP), and 7-high dose (0.60 g/kg of QHP) was 94.89, 74.85, and 77.86%, respectively. QHP at different concentrations had a therapeutic effect on chicken coccidiosis, as the degree of severity of cecal lesions was significantly improved and the presence of bloody feces was reduced in groups medicated with 0.30 and 0.60 g/kg QHP, and both oocyst value and oocyst output were significantly reduced. In addition, the results for rBWG revealed a pattern relatively similar to that of lesion scores, oocyst output and oocyst values among different groups. However, according to the ACI values, the anticoccidial effects of 0.30 g/kg (ACI = 129.16) and 0.60 g/kg (ACI = 144.05) QHP were moderate, and 0.15 g/kg QHP (ACI = 99.37) was insufficient. Research by Del Cacho et al. (36) showed that adding 10 or 17 ppm of artemisinin to the feed significantly affected the formation of oocysts, inhibited the sporulation of oocysts, and reduced cecal damage, but did not affect the formation and development of gametes. Findings from Loredana et al. (19) confirmed that artemisinin supplementation at doses of 5 ppm, 50 ppm, and 500 ppm had prevention and treatment effects on single or mixed infections with *Eimeria acervulina, Eimeria tenella*, and *Eimeria maxima*. The treatments significantly lessened cecum lesions and reduced oocyst output. In this study, the content of artemisinin in QHP was 81.03 mg/g, and the recommended dose was 0.30 g/kg, equivalent to 24-ppm artemisinin. The anticoccidial effect was similar to that of the above-mentioned study.

The lesion score, oocyst value, and ACI value are commonly applied parameters used to evaluate the anticoccidial efficacy of animal drugs. In this study, the ACI values from the drug-treated groups were all <160, which may be related to the virulence of the *E. tenella* strain (Guangdong strain) selected or the concentration of the sporulated oocysts (7 × 10^4^) in the test. Considering that the mortality rate in the infection control group reached 20%, a value that was beyond the optimal range (5–10%) in the experimental design, we can infer that the chicks in the five drug-treated groups were inoculated with a relatively high dose of sporulated *E. tenella* oocysts (37). In addition, the lower ACI value expressed in the toltrazuril control revealed that the *E. tenella* strain used in this study might have high resistance to toltrazuril while having greater sensitivity to SSC. Although the 0.60 g/kg dose of QHP in this experiment had a stronger anticoccidial effect in terms of the lesion score, oocyst value, and survival rate, the cost of choosing 0.60 g/kg is higher, and the research by Yin et al. (17) showed that high-dose artemisinin can have side effects such as neurotoxicity, renal toxicity, and cardiotoxicity. Engberg et al. (38) indicated that the n-hexane extract of *Artemisiae annuae herba* at 0.50 g/kg of dose in feed reduced the food intake and weight gain in chicks. Loredana et al. (19) also confirmed that artemisinin administered continuously for 16 days at a high dose significantly inhibited the body weight gain of chickens. From the above research results, high doses of QHP would be expected to have certain side effects on chicks. Considering that the decrease in body weight gain would also have an economic impact on the poultry breeding industry, a drug dose of QHP at 0.30 g/kg was determined as the recommended dosage and was used for the subsequent target animal safety test to evaluate the long-term effects after 7-day or 14-day continuous administration of QHP. From the results of reduced average weight gain and increased feed conversion rate in chicks in the two QHP-treated groups (0.90 g/kg, 3× RD; 1.80 g/kg, 6× RD) (Table 3) as well as the significant difference in the ROW of liver in the 6× RD group after 14-day administration (Table 4), we conclude that long-term high doses of QHP are likely to be toxic, and that such doses would have an inhibitory effect on the BWG and cause weight loss in chicks.

In this study, except for WBC counts, levels of HGB and MCHC in the 6× RD group, all of the other tested hematological parameters were within the normal range at all stages of the study, and no significant differences were observed between the control and the three treatment groups. The increase in WBC counts may be related to the inflammation induced after hepatocyte injury and to the changes in ALT and TBIL levels (17). RBCs or reticulocytes are sensitive indicators of the toxic effects of artemisinin drugs. *In vivo* animal experiments have found that artemisinins have an inhibitory effect on erythropoiesis. For example, after intravenous administration of artesunate at a dose of 240 mg/kg/d for 3 days, the peripheral blood reticulocytes, RBC counts, and HGB levels in rats were reversibly reduced according to the report by Xie et al. (39). Furthermore, the toxicity of dihydroartemisinin to RBCs is selective or phased, and it mainly affects primitive and young red blood cells. This may affect the cell cycle to inhibit RBC differentiation, as reported by Finaurini et al. (40). The findings of this study indicated the production of circulating white/red blood cells in chicks was not significantly affected by QHP at 3× and 1× the recommend dose levels. In this study, no significant differences in the levels of BUN or CRE in any of the drug groups were noted compared to the control groups; since BUN and CRE serve as confirmatory markers for renal dysfunction and failure (41–43), the above results suggest that 14-day continuous administration of QHP had little negative impact on the kidney function of chicks. As for the biochemical parameters, ALT and AST are well-known as markers of cell damage, especially hepatocyte necrosis (44–46). Moreover, TBIL (the product of hemoglobin degradation) is an important indicator and sign of liver damage and cholestasis and is also related to increased hemolysis (47–49). In our assay, the levels of ALT, AST, and TBIL in the 6× RD and 3× RD groups increased significantly after seven days of administration (P < 0.05); however, at the 14th day after administration, the concentrations of ALT and AST returned to normal levels, indicating that the increase in the levels of these two enzymes may be related to the reversible damage of liver cells stimulated by high artemisinin concentrations. Moreover, this signified that QHP has moderate toxic effects on chicks after 14 days of administration at a daily dose above the 0.60 g/kg recommended dose. However, the macroscopic examinations of the organs of chicks in the three QHP-treated groups produced no apparent changes compared with the control groups, and the necropsy results were not in agreement with the hematological and serum biochemical analyses. Moreover, these findings were not confirmed or supported by the histopathological analysis of the livers and kidneys, where QHP did not show toxic effects on the vital organs, and there was no abnormal tissue damage in the three QHP-treated groups. In conclusion, the present study recommends a dose of 0.30 g/kg feed of QHP, as this is likely to be non-toxic.

## Conclusion

In this study, the content of artemisinin in QHP was 81.03 mg/g, and the addition of QHP (0.30 and 0.60 g/kg feed) could increase the rBWG and survival rate of broiler chicks infected with *E. tenella* (Guangdong strain) while reducing bloody diarrhea, oocyst output, and lesion scores in the cecal region. The ACI values in 0.30 and 0.60 g/kg QHP-treated groups were between those of toltrazuril and SSC treatments, indicating that QHP had prevention and treatment effects in chicks. From the safety test, a dose of 0.30 g/kg feed of this plant-derived anticoccidial was recommended as it presented no QHP-related signs of toxicity or abnormalities in target animal safety tests. Findings from this study provided information for designing new plant-based drug against coccidiosis infection. Therefore, the dosage in clinical applications should be set according to the recommended dose to ensure animal safety, and QHP at a dose of 0.30 g/kg feed would be appropriate for the therapy and intermittent treatment of *E. tenella*-infected chicks.

## Data Availability Statement

The original contributions presented in the study are included in the article/supplementary material, further inquiries can be directed to the corresponding authors.

## Ethics Statement

The animal study was reviewed and approved by the Ethics Committee of Lanzhou Institute of Husbandry and Pharmaceutical Sciences of the Chinese Academy of Agricultural Sciences.

## Author Contributions

LW: development of methodology, analysis and interpretation of data, and writing of the manuscript. WZG: performed the experiments and coordinated and supervised the study. SUH: acquisition of data and writing of the manuscript. ZTG: conception and design and provided background information. DAC: acquisition and analysis of data and development of methodology. FY: analyzed and interpreted the references and material support. FC: development of methodology and analysis and interpretation of data. XJW: preparation of serum samples and material support. JWL: provided background information and references amended. All authors contributed to the article and approved the submitted version.

## Funding

This work was supported by Agricultural Science and Technology Innovation Program of Chinese Academy of Agricultural Sciences (grant No. CAAS-ASTIP-2014-LIHPS-04), Major Output Scientific Research Items of Chinese Academy of Agricultural Sciences (grant No. CAAS-ZDXT2018008-4), Special Fund of the Chinese Central Government for Basic Scientific Research Operations in Commonweal Research Institutes (grant No. 1610322020005), and Natural Science Foundation of Gansu Province (grant No. 18JR3RA398).

## Conflict of Interest

The authors declare that the research was conducted in the absence of any commercial or financial relationships that could be construed as a potential conflict of interest.

## Publisher's Note

All claims expressed in this article are solely those of the authors and do not necessarily represent those of their affiliated organizations, or those of the publisher, the editors and the reviewers. Any product that may be evaluated in this article, or claim that may be made by its manufacturer, is not guaranteed or endorsed by the publisher.

## Abbreviations

QHP: Qinghao Powder
HPLC: high-performance liquid chromatography
TLC: thin-layer chromatography
PI: post infection
HC: healthy control group
IC: infected control group
TC: toltrazuril control
SS: sulfachloropyrazine sodium
SSC: sulfachloropyrazine sodium control
BWG: body weight gain
rBWG: relative body weight gain
ACI: anticoccidial index
RBC: red blood cells
HGB: hemoglobin concentration
HCT: hematocrit
MCHC: mean corpuscular hemoglobin concentration
WBC: white blood cell
Lym: lymphocyte
ML: monocytes
ALT: alanine aminotransferase
AST: aspartate aminotransferase
TP: total protein
TBIL: total bilirubin
BUN: blood urea nitrogen
CER: creatinine.
